# Predictors for metamorphopsia in eyes undergoing macular hole surgery

**DOI:** 10.1038/s41598-023-28031-2

**Published:** 2023-01-16

**Authors:** Asuka Takeyama, Yutaka Imamura, Taichi Fujimoto, Toshiya Iida, Yuko Komiya, Masaki Shibata, Masahiro Ishida

**Affiliations:** 1 Department of Ophthalmology, Toho University Ohashi Medical Center, 2–22–36, Ohashi, Meguro-Ku, Tokyo, 153–8515 Japan; 2 Department of Ophthalmology, Teikyo University School of Medicine, University Hospital Mizonokuchi, 5-1-1, Futako, Takatsu-ku, Kawasaki City, Kanagawa 213-8507 Japan

**Keywords:** Retinal diseases, Predictive markers

## Abstract

Metamorphopsia is an important visual symptom of macular disease. We determined predictors for metamorphopsia investigating the relationships of macular hole (MH) diameter and retinal layer thicknesses with metamorphopsia after MH surgery. Forty-two eyes of 42 consecutive patients undergoing MH surgery were retrospectively studied. Metamorphopsia was measured with M-CHARTS. Inner nuclear layer (INL) and outer retinal layer (OR) thicknesses were measured 1000 μm away from central fovea at using Spectralis. Preoperative M-CHARTS scores correlated with MH diameters (P = 0.007–0.031) and changes of temporal OR thickness (P = 0.008–0.010). Postoperative M-CHARTS score at 3 months correlated with preoperative nasal and inferior OR thicknesses (P = 0.003 and 0.016) and with changes of superior INL at 3 and 6 months (P = 0.011 and 0.025), and score at 1 month with change of temporal OR at 6 months (P = 0.033). Postoperative improvement of M-CHARTS scores correlated with changes of temporal INL and superior OR (P = 0.026 and 0.002). Multiple regression analysis revealed that MH diameter was a significant predictor for metamorphopsia. Photoreceptor displacement and inner retinal change may generate metamorphopsia in MH undergoing surgery, however MH diameter is the most powerful predictor.

## Introduction

Metamorphopsia is an important visual symptom of macular disease. Patients with macular hole (MH) complain of metamorphopsia before and after surgery. Anatomic closure rates and functional outcomes of MH have improved with advances in surgical techniques, including techniques for manipulation of the internal limiting membrane (ILM)^1–3^. Even after successful MH surgery residual metamorphopsia affects the patients’ daily life^4^.

Metamorphopsia before and after MH surgery was quantified using M-CHARTS^5^, and parameters of optical coherence tomography (OCT) have been related with metamorphopsia scores^6–9^. However, the exact mechanism of metamorphopsia in MH is not clear.

Earlier, we reported that ILM peeling in MH surgery made the flexible retina retract toward the optic disc, and that retinal displacement of temporal retinal vessels after surgery correlated with basal diameter of MH^10^. Total retinal thickness after MH surgery decreased in all sectors except for the nasal^11^, and retinal displacement correlated with the change of inner nuclear layer (INL) thickness^12^, which indirectly supporting our hypothesis that retinal displacement was caused by the contraction of optic nerve fibers^10–12^. Photoreceptor layers move to close MH and inner retina shrinks toward the optic disc possibly due to contraction of optic nerve fibers. Therefore, dynamic changes of retinal structure occur during the closure of MH.

As a first step toward understanding the mechanism of metamorphopsia in MH, we analyzed the relationships of metamorphopsia scores with retinal structural parameters including MH diameters, distances of retinal displacement, and retinal layer thicknesses.

## Methods

This was a retrospective, consecutive, case series study. Patients’ selection, ophthalmic examination, and surgical procedures were previously reported in detail^12^. Briefly, patients who were able to follow up at least 6 months postoperatively were included in this study. All patients underwent examinations at 1, 3 and 6 months postoperatively. Eyes with an axial length of 27 mm or longer, a history of retinal surgery and ocular diseases such as diabetic retinopathy, rhegmatogenous retinal detachment, uveitis or ocular trauma, and those undergoing inverted ILM flap technique were excluded.

This study was approved by the Institutional Review Board of Teikyo University School of Medicine (No.20–206) and conformed to the tenets of the Declaration of Helsinki. Due to the retrospective nature of the study, need to obtain informed consent was waived by the Institutional Review Board of Teikyo University School of Medicine.

Three vitreoretinal surgeons (M.I., Y.I. and A.T.) performed MH surgeries from March 2017 to July 2020 at the Department of Ophthalmology, Teikyo University School of Medicine, University Hospital Mizonokuchi (Kanagawa, JAPAN). All patients underwent a standard 3-port PPV with 25-gauge instruments and combined ILM peeling using the 0.25 mg/ml brilliant blue G (BBG) or 10 mg/ml triamcinolone acetonide (TA). The ILM peeling was performed in all quadrants, to the edge of the vascular arcade and close to the optic nerve papillary margin for the nasal part. After air-fluid exchange, intravitreal air was replaced with 20% sulfur hexafluoride. Details of surgical procedures were reported previously^12^.

**Figure 1:**
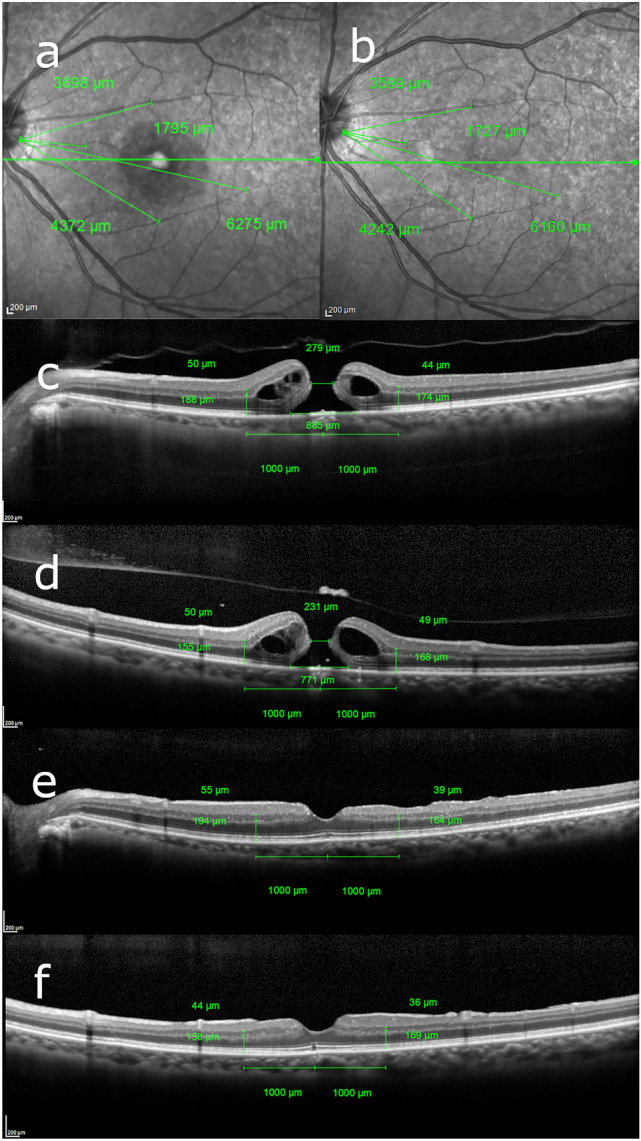
Measurement of optical coherence tomography parameters in macular hole using Spectralis. (**a**) Near-infrared images of Spectralis optical coherence tomography were used to measure the retinal distance between the temporal margin of the optic disc and the intersections or bifurcations of retinal vessels. Four intersections or bifurcations of retinal blood vessels in the temporal, nasal, superior and inferior the Early Treatment Diabetic Retinopathy Study subfields were selected. (**b**) Retinal distances at 6 months postoperatively were measured with the same methods. (**c**) In horizontal scan images, the horizontal minimum and basal diameters of the MH were measured. Inner nuclear layer (INL) and outer retinal layer (OR) thicknesses were measured 1000 μm away from the center of MH temporally and nasally. (**d**) In vertical scan images, the vertical minimum and basal diameters of MH were measured. The INL and OR thicknesses were measured with as was done for horizontal images. The right side of the image is superior retina, and the left is inferior. (**e**) Retinal layer thicknesses in the horizontal scan image at 1 month postoperatively. (**f**) Retinal layer thicknesses in the superior and inferior sectors in the vertical scan image at 6 months postoperatively.

The basal and minimum MH diameters in the vertical and horizontal directions, retinal thickness and retinal distance were manually measured with spectral-domain OCT (Spectralis; Heidelberg Engineering, Inc., Heidelberg, Germany) images using caliper function. The methods of selecting sectors and measuring retinal thickness with Spectralis OCT images were described previously^12^. We measured each retinal layer 1000 μm away from the center of the fovea in four sections of macular: the nasal, temporal, superior and inferior quadrants in order to get rid of cystic change around the fovea as reported previously^13^. INL thickness and outer retinal layer (OR) thickness in each sector were measured manually using the caliper function of Spectralis OCT. Similar to our previous report^12^, the distance between the temporal margin of the optic disc and an intersection or bifurcation of retinal vessels was manually measured in four sectors on near-infrared images, and the rates of change in retinal distance after surgery was used for statistical analysis as retinal displacement (%). Measurement of the MH diameters, retinal distance and retinal thickness are shown in Fig. 1. The retinal thicknesses and retinal displacements (%) in four sectors were measured preoperatively and at 1, 3 and 6 months postoperatively.

The best-corrected visual acuity (BCVA) was measured using a Landolt C chart at each visit and was converted to the logarithm of the minimal angle of resolution (logMAR) units for statistical analyses. M-CHARTS (Inami Co., Tokyo, Japan) was used to quantify the degree of metamorphopsia for both vertical and horizontal lines. The detailed procedure for M-CHARTS scoring is described in our previous reports^14^. The BCVA and M-CHARTS scores were measured at each visit.

Normality of the data was evaluated using the Shapiro–Wilk test, and a nonparametric test was selected because the data did not follow a normal distribution. The Wilcoxon signed-rank test was used to evaluate comparisons between preoperative and postoperative M-CHARTS scores. Spearman’s rank correlation coefficient test was used to determine correlations between M-CHARTS score and MH diameter, retinal displacement (%) and changes of retinal thickness. Multiple regression analysis was performed to identify independent parameters associated with metamorphopsia. Statistical analyses were analyzed with SPSS software version 24.0 (SPSS, Chicago, Illinois, USA). A difference was considered to be statistically significant at P < 0.05.

## Results

Forty-two eyes of 42 patients (20 women) were studied (aged 66.6 ± 5.8 years [mean ± standard deviation]; age range 51–78 years). Fourteen eyes (33.3%) were classified as Stage 2 MH, 18 eyes (42.9%) as Stage 3, and 10 eyes (23.8%) as Stage 4.

Forty-one phakic eyes were performed phacoemulsification with intraocular lens implantation simultaneously with vitrectomy. During ILM peeling, BBG was used for 28 eyes and TA for 14 eyes. MHs were closed in all the eyes after initial surgery.

The average horizontal minimum MH diameter was 273.9 ± 111.6 μm (range 106–584 μm) and the vertical minimum MH diameter was 233.7 ± 102.3 μm (range 51–513 μm), whereas the average horizontal basal MH diameter was 670.3 ± 235.9 μm (range 109–1108 μm) and the vertical basal MH diameter was 582.6 ± 224.3 μm (range 109–983 μm).

The BCVA was 0.57 ± 0.28 logMAR units preoperatively, 0.22 ± 0.19 logMAR units at 1 month, 0.13 ± 0.17 logMAR units at 3 months, 0.11 ± 0.17 logMAR units at 6 months postoperatively. The average BCVA improved gradually and significantly for 6 months postoperatively (P value range: P < 0.001–0.017).

The preoperative BCVA did not correlate with the vertical and horizontal M-CHARTS scores (P = 0.529 and 0.417). The BCVA was significantly correlated with the vertical M-CHARTS score at 1 month postoperatively (r = 0.340, P = 0.030), but not at the other visits.

### Time course of average M-CHARTS scores

The average preoperative vertical M-CHARTS score was 0.89 ± 0.56, and improved to 0.53 ± 0.42 at 1 month, 0.51 ± 0.43 at 3 months and 0.49 ± 0.45 at 6 months (all P < 0.001). The average preoperative horizontal M-CHARTS score was 0.61 ± 0.39, which improved to 0.36 ± 0.41 at 1 month (P < 0.001), 0.33 ± 0.29 at 3 months (P < 0.001) and 0.38 ± 0.39 at 6 months postoperatively (P = 0.007).

### Correlations of vertical and horizontal M-CHARTS scores with the size of macular hole

Correlations of pre- and postoperative M-CHARTS scores with horizontal and vertical MH diameters are shown in Table 1. The preoperative horizontal and vertical M-CHARTS scores showed significant correlations with the horizontal minimum, horizontal basal, and vertical basal MH diameters. The horizontal and vertical M-CHARTS scores at 3 months correlated with the vertical and horizontal basal MH diameters (r and P value range: r = 0.394–0.401, P = 0.009–0.010), and the horizontal M-CHARTS score at 6 months correlated with the horizontal minimum MH diameter (r = 0.309, P = 0.049). The horizontal and vertical M-CHARTS scores at 1 month did not correlate with any of MH diameters.

**Table 1:** Correlations of pre- and postoperative M-CHARTS score with the horizontal and vertical diameter of the macular hole.

	Horizontal				Vertical			
	Basal diameter		Minimum diameter		Basal diameter		Minimum diameter	
	r	P	r	P	r	P	r	P
Baseline								
MH	**0.409**	0.007*	**0.378**	0.014*	**0.376**	0.014*	0.242	0.122
MV	**0.350**	0.023*	**0.334**	0.031*	**0.353**	0.022*	0.172	0.277
At 3 months								
MH	**0.395**	0.010*	**0.428**	0.005*	**0.401**	0.009*	0.237	0.130
MV	**0.394**	0.010*	0.268	0.086	**0.381**	0.010*	0.139	0.379
At 6 months								
MH	0.205	0.198	**0.309**	0.049*	0.203	0.204	0.107	0.505
MV	0.247	0.120	0.275	0.082	0.288	0.068	0.134	0.403

Spearman’s rank correlation coefficient was used to determine the significance of the correlations.

*MH* horizontal score of M-CHARTS, *MV* vertical score of M-CHARTS.

Significant P value are indicated by asterisk (*).

Significant values are in bold.

### Correlations of M-CHARTS scores with retinal displacement

Preoperative vertical and horizontal M-CHARTS scores did not correlate with the retinal displacement (%) in any of four sections, and postoperative M-CHARTS scores did not correlate with the retinal displacement (%) at any visits (all P values > 0.05).

### Correlations of M-CHARTS scores with pre- and postoperative changes in inner nuclear layer and outer retinal layer thicknesses

Correlations between M-CHARTS scores and preoperative INL and OR thickness are shown in Table 2. Preoperative M-CHARTS score showed a positive correlation with preoperative temporal OR thickness. Postoperative M-CHARTS scores correlated with preoperative nasal, temporal and inferior OR thicknesses.

**Table 2:** Correlations between M-CHARTS scores and preoperative inner nuclear layer and outer retinal thickness.

	Baseline				At 1 month				At 3 months				At 6 months			
	MH		MV		MH		MV		MH		MV		MH		MV	
	r	P	r	P	r	P	r	P	r	P	r	P	r	P	r	P
Baseline																
Nasal INL	0.253	0.106	0.152	0.338	– 0.023	0.885	0.051	0.753	0.031	0.846	0.210	0.183	– 0.136	0.396	– 0.067	0.677
Nasal OR	0.153	0.334	0.290	0.063	**0.327**	0.037*	0.099	0.539	**0.445**	0.003*	0.265	0.090	**0.338**	0.031*	0.247	0.120
Temporal INL	0.191	0.225	0.059	0.708	– 0.074	0.645	– 0.028	0.863	– 0.008	0.959	0.196	0.213	– 0.171	0.285	– 0.074	0.645
Temporal OR	0.295	0.058	**0.306**	0.049*	**0.356**	0.022*	0.239	0.133	**0.326**	0.035*	**0.307**	0.048*	0.168	0.295	0.215	0.176
Superior INL	0.229	0.145	0.180	0.253	– 0.078	0.626	0.106	0.511	0.076	0.634	0.234	0.137	0.027	0.868	0.014	0.931
Superior OR	0.224	0.153	0.116	0.465	0.231	0.146	– 0.085	0.598	0.274	0.080	0.114	0.472	0.133	0.405	0.216	0.175
Inferior INL	0.166	0.295	0.169	0.284	0.041	0.799	– 0.109	0.498	0.124	0.434	0.118	0.457	– 0.005	0.976	– 0.023	0.888
Inferior OR	0.137	0.387	0.085	0.591	0.250	0.115	0.224	0.159	**0.310**	0.046*	**0.369**	0.016*	0.263	0.097	**0.367**	0.018*

Spearman’s rank correlation coefficient was used to determine the significance of the correlations.

*MH* horizontal score of M-CHARTS, *MV* vertical score of M-CHARTS, *INL* inner nuclear layer thickness, *OR* outer retinal thickness.

Significant P value are indicated by asterisk (*).

Significant values are in bold.

Correlations between M-CHARTS scores and the rates of change in INL and OR thickness are shown in Table 3. Preoperative M-CHARTS score did not correlate with the rate of change in INL thickness at any visits. There were significant correlations between preoperative M-CHARTS scores and the rates of change in temporal OR thicknesses at all postoperative visits. Postoperative vertical M-CHARTS score at 1 month correlated with the change of superior INL thickness at 1 month and that of temporal OR thickness at 3 and 6 months, and vertical M-CHARTS score at 3 months with that of superior INL at 3 months, indicating that both inner and outer retinal structural changes after MH closure may affect postoperative metamorphopsia.

**Table 3:** Correlations between M-CHARTS scores and rates of change of inner nuclear layer and outer retinal thickness.

	Baseline				At 1 month				At 1 month				At 6 months\			
	MH		MV		MH		MV		MH		MV		MH		MV	
	r	P	r	P	r	P	r	P	r	P	r	P	r	P	r	P
At 1 month																
Nasal INL%	– 0.171	0.280	– 0.117	0.461	0.079	0.622	– 0.029	0.859	0.065	0.681	– 0.136	0.390	0.251	0.113	0.173	0.279
Nasal OR%	– 0.104	0.513	– 0.235	0.134	– 0.058	0.719	– 0.028	0.862	– 0.256	0.102	– 0.246	0.117	– 0.203	0.204	– 0.087	0.589
Temporal INL%	– 0.254	0.104	– 0.084	0.596	0.102	0.526	– 0.025	0.876	– 0.030	0.851	– 0.292	0.060	0.072	0.655	– 0.077	0.631
Temporal OR%	**– 0.321**	0.038*	**– 0.405**	0.008*	– 0.181	0.257	– 0.305	0.053	– 0.269	0.085	– 0.207	0.188	– 0.116	0.469	– 0.150	0.349
Superior INL%	– 0.213	0.176	– 0.234	0.136	– 0.085	0.599	**– 0.319**	0.042*	– 0.174	0.272	**– 0.309**	0.046*	– 0.069	0.670	– 0.119	0.457
Superior OR%	– 0.205	0.193	– 0.044	0.780	0.027	0.869	0.268	0.090	– 0.053	0.739	0.151	0.341	0.066	0.682	0.050	0.757
Inferior INL%	– 0.170	0.283	– 0.205	0.193	0.047	0.769	0.059	0.714	– 0.067	0.673	– 0.148	0.350	0.007	0.968	0.019	0.907
Inferior OR%	– 0.298	0.056	– 0.165	0.298	– 0.127	0.430	– 0.154	0.338	– 0.329	0.033	– 0.279	0.074	– 0.228	0.152	– 0.236	0.137
At 3 months																
Nasal INL%	– 0.191	0.227	– 0.162	0.304	– 0.006	0.969	– 0.057	0.725	0.023	0.886	– 0.228	0.147	0.202	0.205	0.044	0.783
Nasal OR%	– 0.063	0.694	– 0.170	0.282	0.010	0.953	0.024	0.883	– 0.148	0.349	0.072	0.237	– 0.108	0.501	– 0.046	0.775
Temporal INL%	– 0.071	0.653	– 0.052	0.745	0.077	0.633	– 0.087	0.587	– 0.067	0.674	– 0.187	0.234	0.134	0.404	– 0.013	0.938
Temporal OR%	– 0.296	0.057	**– 0.392**	0.010*	– 0.137	0.392	**– 0.312**	0.047*	– 0.067	0.674	– 0.187	0.234	0.075	0.640	– 0.032	0.842
Superior INL%	– 0.203	0.198	– 0.206	0.191	– 0.090	0.575	– 0.297	0.059	– 0.119	0.452	**– 0.387**	0.011*	– 0.110	0.493	– 0.286	0.070
Superior OR%	– 0.247	0.116	– 0.121	0.444	– 0.066	0.683	0.143	0.374	– 0.094	0.554	0.082	0.605	0.091	0.573	0.071	0.661
Inferior INL%	– 0.113	0.476	– 0.208	0.185	0.016	0.921	0.114	0.478	– 0.042	0.792	– 0.114	0.470	– 0.023	0.887	– 0.046	0.774
Inferior OR%	– 0.299	0.054	– 0.195	0.215	– 0.121	0.450	– 0.167	0.296	– 0.217	0.167	– 0.235	0.135	– 0.118	0.463	– 0.212	0.183
At 6 months																
Nasal INL%	– 0.216	0.170	– 0.158	0.317	0.067	0.678	– 0.027	0.869	0.081	0.609	– 0.153	0.333	0.236	0.137	0.111	0.492
Nasal OR%	– 0.136	0.392	– 0.198	0.209	– 0.108	0.501	– 0.132	0.409	– 0.232	0.140	– 0.262	0.093	– 0.137	0.392	– 0.160	0.316
Temporal INL%	0.072	0.650	0.068	0.670	0.221	0.165	0.127	0.428	0.178	0.260	– 0.047	0.769	0.231	0.146	0.084	0.601
Temporal OR%	– 0.285	0.067	**– 0.398**	0.009*	– 0.130	0.418	**– 0.335**	0.033*	– 0.103	0.516	– 0.235	0.134	– 0.004	0.979	– 0.133	0.407
Superior INL%	– 0.282	0.070	– 0.194	0.217	0.055	0.730	– 0.253	0.110	– 0.020	0.899	**– 0.346**	0.025*	0.005	0.977	– 0.089	0.581
Superior OR%	– 0.131	0.407	– 0.057	0.722	– 0.161	0.315	0.161	0.315	– 0.131	0.407	0.102	0.520	0.027	0.868	– 0.053	0.743
Inferior INL%	– 0.082	0.607	– 0.148	0.350	0.033	0.838	0.158	0.323	– 0.030	0.852	– 0.098	0.537	0.005	0.974	0.011	0.945
Inferior OR%	– 0.246	0.116	– 0.200	0.204	– 0.161	0.315	– 0.177	0.268	– 0.260	0.097	– 0.279	0.074	– 0.088	0.585	– 0.123	0.443

INL% and OR% were defined as the rates of change before and after surgery in INL and OR thicknesses measured in four sections of the macula: nasal, temporal, superior, and inferior, at 1000 μm away from the center of the fovea.

Spearman’s rank correlation coefficient was used to determine the significance of the correlations.

*MH* horizontal score of M-CHARTS, *MV* vertical score of M-CHARTS, *INL* inner nuclear layer thickness, *OR* outer retinal thickness, *INL* % the rate of change in inner nuclear layer thickness, *OR* % the rate of change in outer retinal thickness.

Significant P value are indicated by asterisk (*).

Significant values are in bold.

### Correlation between pre- and postoperative M-CHARTS score differences and changes in inner nuclear layer and outer retinal layer thickness

**Figure 2:**
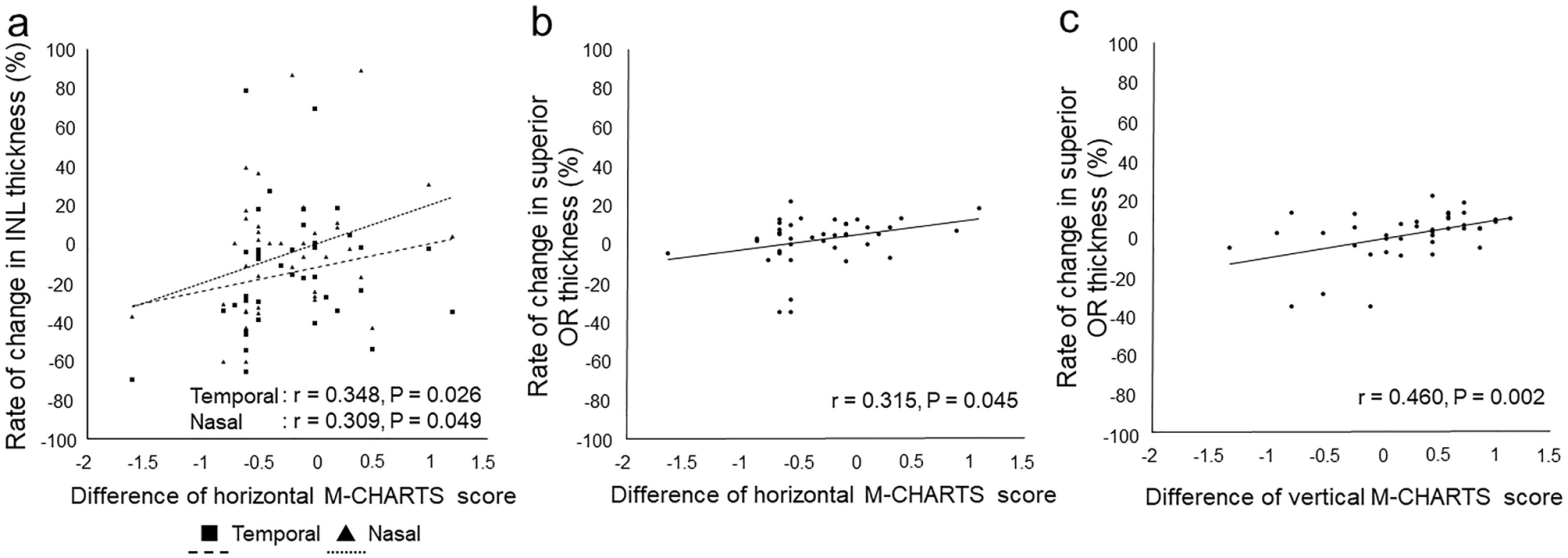
Correlations between pre- and postoperative differences in M-CHARTS score and changes in inner nuclear layer and outer retinal layer thickness at 1 month postoperatively. Plots shows the correlations between difference in horizontal M-CHARTS score and rate of change in temporal and superior inner nuclear layer thickness (**a**) and the rate of change in superior outer retinal layer (OR) thickness (**b**), and the difference in vertical M-CHARTS score correlated with the rate of change in superior OR thickness (**c**) at 1 month postoperatively. Spearman’s rank correlation coefficient was used to determine the significance of the correlations.

The correlations between the pre- and postoperative differences in M-CHARTS score and the rates of change in retinal thickness at 1 month postoperatively are shown in Fig. 2. The difference in horizontal M-CHARTS score correlated with the rates of change in nasal and temporal INL thickness (r = 0.309, P = 0.049 and r = 0.348, P = 0.026) and the rate of change in superior OR thickness at 1 month postoperatively (r = 0.315, P = 0.045), and the difference in vertical M-CHARTS score correlated with the rate of change in superior OR thickness at 1 month postoperatively (r = 0.460, P = 0.002).

The difference in horizontal M-CHARTS score at 6 months correlated with the rate of change in superior OR thickness at 3 months postoperatively (r = 0.315, P = 0.042), and the difference in vertical M-CHARTS at 6 months correlated with the rate of change in temporal OR thickness at 1 and 3 months postoperatively (r = 0.304, P = 0.050 and r = 0.369, P = 0.016).

Thus, the greater the postoperative change of INL and OR thickness, the greater the improvement of metamorphopsia after MH surgery.

### Correlations of basal macular hole diameters with preoperative inner nuclear layer and outer retinal layer thicknesses

The average INL thickness in each of the four sections at baseline was positively correlated with the vertical and horizontal basal MH diameters (r and P value range: r = 0.358–0.587, P < 0.001–0.020). The average OR thickness in the nasal and temporal sections at baseline were correlated with the vertical and horizontal basal MH diameters (r and P value range: r = 0.384–0.416, P = 0.006–0.012).

### Multivariate analysis to identify predictors of the M-CHARTS score

Multiple regression analysis was performed to identify predictors for the M-CHARTS scores at baseline and 6 months postoperatively. The dependent factors were defined as the vertical and horizontal M-CHARTS scores at baseline and 6 months postoperatively, and the independent factors defined as age, sex, rates of change in INL and OR thickness, retinal displacement (%) and basal MH diameter. The rates of change in INL and OR thickness and retinal displacement (%) for the nasal, temporal, superior and inferior sectors were selected respectively. Because vertical and horizontal basal MH diameters were strongly correlated (r = 0.961, P < 0.001, Spearman’s rank correlation coefficient test), we used horizontal basal MH diameters and vertical basal MH diameters separately in different models. As shown in Tables 4 and 5, larger MH diameter is an only significant predictive factor for the higher M-CHARTS scores at baseline and 6 months postoperatively (P value range: P = 0.007–0.049).

**Table 4:** Multivariate regression analysis for M-CHARTS score at baseline.

	Coefficient	SE	95% CI	t	P
Dependent factor: horizontal M-CHARTS					
Independent factors on the nasal sector					
Horizontal MH diameter	0.436	< 0.001	< 0.001–0.002	2.863	0.007
Dependent factor: horizontal M-CHARTS					
Independent factors on the temporal sector					
Horizontal MH diameter	0.405	< 0.001	− 0.012–0.016	2.264	0.013

Independent factors: age, sex, INL%, OR%, RD%, MH diameter.

The INL%, OR% and RD% for the nasal, temporal, superior and inferior sectors were selected respectively.

Horizontal MH diameter was selected for the nasal and temporal sectors and vertical MH diameter was selected for the superior and inferior sectors.

INL% and OR% were defined as the rates of change before and after surgery in INL and OR thicknesses measured in four sections of the macula: nasal, temporal, superior, and inferior, at 1000 μm away from the center of the fovea.

*SE* standard error, *CI* confidence Interval, *MH* macular hole, *INL*% the rate of change in inner nuclear layer thickness, *OR*% the rate of change in outer retinal layer thickness, *RD* % the rate of change in retinal distance.

**Table 5:** Multivariate regression analysis for M-CHARTS score at 6 months after surgery.

	Coefficient	SE	95% CI	t	P
Dependent factor: vertical M-CHARTS score					
Independent factors on superior sector					
Vertical MH diameter	0.331	< 0.001	< 0.001–0.002	2.049	0.045
Independent factor on inferior sector					
Vertical MH diameter	0.325	< 0.001	< 0.001–0.002	2.076	0.049
Independent factor on nasal sector					
Horizontal MH diameter	0.334	< 0.001	< 0.001–0.002	2.205	0.043
Dependent factor: horizontal M-CHARTS score					
Independent factor on nasal sector					
Horizontal MH diameter	0.436	< 0.001	< 0.001–0.002	2.863	0.007
Independent factor on temporal sector					
Horizontal MH diameter	0.405	< 0.001	< 0.001–0.002	2.624	0.013

Independent factors: age, sex, INL%, OR%, RD%, MH diameter.

The INL%, OR% and RD% for the nasal, temporal, superior and inferior sectors were selected respectively.

Horizontal MH diameter was selected for the nasal and temporal sectors and vertical MH diameter was selected for the superior and inferior sectors.

INL% and OR% were defined as the rate of change before and after surgery in INL and OR thicknesses measured in four sections of the macula: nasal, temporal, superior, and inferior, at 1000 μm away from the center of the fovea.

*SE* standard error, *CI* confidence Interval, *MH* macular hole, *INL*% the rate of change in inner nuclear layer thickness, *OR*% the rate of change in outer retinal layer thickness, *RD %* the rate of change in retinal distance.

## Discussion

Our results showed that metamorphopsia at baseline correlated with the MH diameters and changes in temporal OR thickness. Patients with greater degree of postoperative thinning of the superior INL at 3 and 6 months and temporal OR at 6 months had greater degree of postoperative metamorphopsia. Improvement of the M-CHARTS scores correlated with the rates of changes in INL and OR thickness at 1 month, indicating that the greater the degree of postoperative thinning of the INL and OR thickness, the greater the improvement in postoperative metamorphopsia. Multiple regression analysis showed that MH diameter significantly influenced the pre- and postoperative M-CHARTS scores, and that the MH diameter was the most important factor influencing the MH metamorphopsia. Retinal displacement occurring after MH surgery did not correlate with metamorphopsia.

We have previously reported that the change in INL thickness significantly correlates with retinal displacement after ILM peeling, and the temporal retinal displacement correlates with the basal MH diameter^12^. The retina moves toward the optic disc during MH closure and we found that distance of retinal displacement correlated with the change in the INL thickness, but not the changes in OR thickness^12^. Dynamic structural changes of the inner retina occur during MH closure, which appears to generate part of postoperative metamorphopsia after MH surgery.

The mechanism by which retinal deformation causes metamorphopsia has been discussed in previous publications. Asymmetric elongation of the foveal tissue was associated with postoperative metamorphopsia in eyes undergoing MH surgery^8^. Park et al.^9^ reported that a large extent of ILM detachment caused the square grid on the retina to move towards the optic nerve and postoperative metamorphopsia corelated with parafoveal deformation. The authors considered that metamorphopsia after MH surgery was caused by irregularities and eccentric displacement of the photoreceptor layer^15,16^. Sugiura et al.^6^ reported that the area of the intraretinal cyst was most associated with metamorphopsia. It is interesting that their study did not show a positive association between metamorphopsia and MH diameter. Probably their measurement may have represented dislocation of photoreceptors more than MH diameter among the patients they collected in their study.

Earlier, our team reported that INL is a biomarker of metamorphopsia in patients with epiretinal membrane (ERM)^17^. Experimental studies showed that Müller cell is an optic fiber which transfers photons to photoreceptors^18,19^, and we considered that dislocated Müller cells transfer photons to the photoreceptors which located away from original positions, which creates a sensation of metamorphopsia^17^. After MH surgery, changes in INL thickness correlated with changes in metamorphopsia, suggesting that Müller cells play a role for sensation of metamorphopsia in eyes undergoing MH surgery. A shrinkage of the INL caused by retinal movement in eyes with larger MH appears to create greater postoperative scores of M-CHARTS. However, before MH surgery, MH diameter is an important parameter to determine the degree of metamorphopsia. The preoperative INL and OR thicknesses were influenced by the size of the MH, of which the baseline OR thickness correlated the pre- and postoperative metamorphopsia. Multiple regression analysis showed that the MH diameter is a predictor for the postoperative M-CHARTS score, which indicates that morphological changes in the photoreceptor layer associated with MH has great influence on postoperative metamorphopsia. In contrast, the M-CHARTS score did not correlate with retinal displacement, indicating that the displacement of retina existing relatively far away from the central fovea did not affect metamorphopsia. This means that the displacement of photoreceptor cells near the central fovea during MH closure appears to play the most important role in postoperative metamorphopsia.

**Figure 3:**
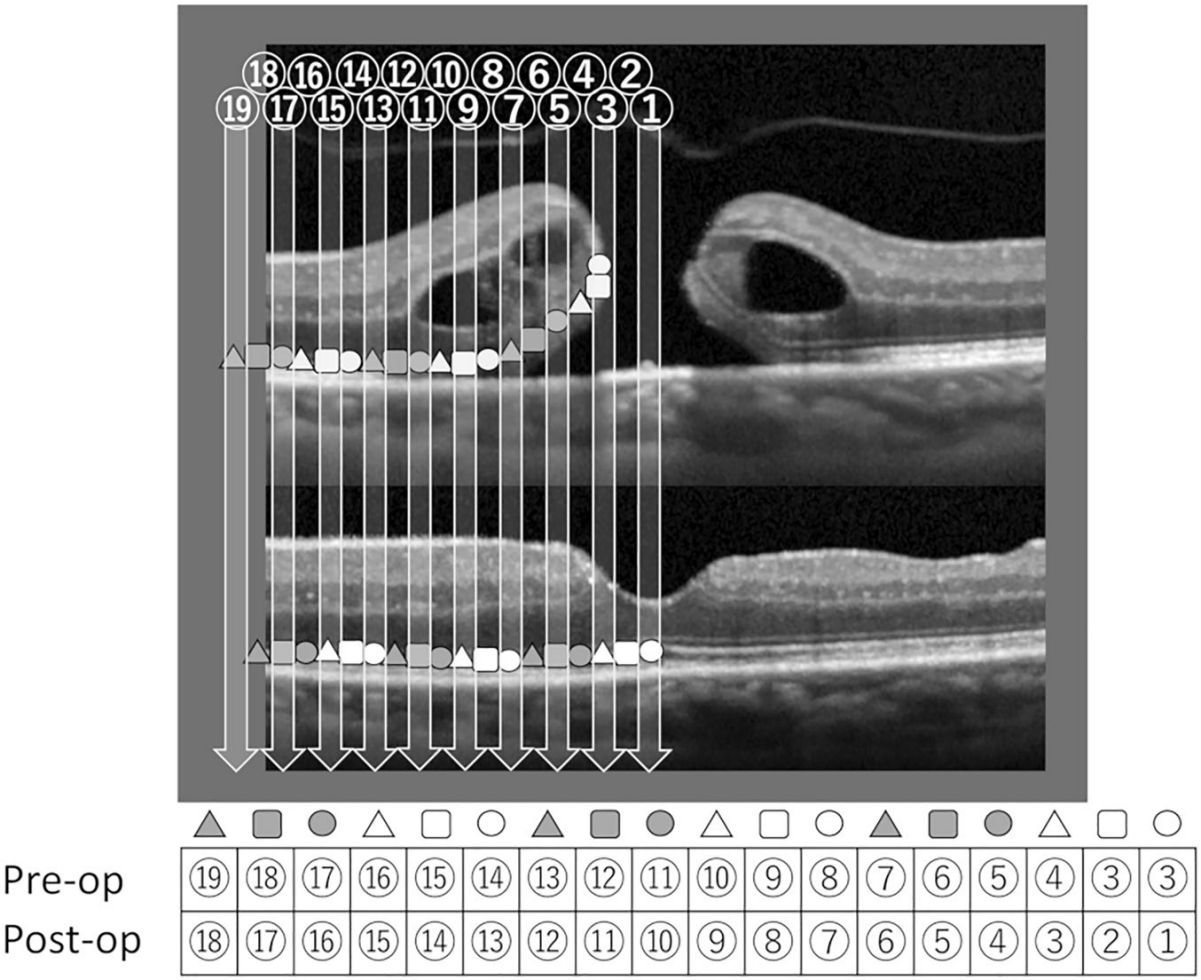
Schematic diagram and optical coherence tomography image showing the mechanism of metamorphopsia before and after macular hole surgery. The photoreceptor cells near the center of the macula are detached from retinal pigment epithelium and efferently displaced. There are no photoreceptor cells that receive light from light sources ① and ② in the macular hole (MH) and there are two photoreceptor cells that receive light from light source ③. After the closure of the MH, the photoreceptor cells are slightly displaced from their original position, which generates metamorphopsia postoperatively. Dislocated Müller cells may also contribute to generate metamorphopsia, working as optic guide to transfer photons to dislocated photoreceptors.

The mechanism of metamorphopsia in patients with MH is explained using OCT images and schematic diagrams in Fig. 3. The photoreceptor cells near the center of the macular with the whole retinal layer are efferently displaced during the formation of the MH, detached from the retinal pigment epithelial cells, and elevated toward the vitreous. As a result, the density of photoreceptor cells increases along the optical axis and the overlapping photoreceptor cells are simultaneously stimulated with incoming light, causing metamorphopsia in which percepted images pinch toward the center. Early after MH closure, photoreceptor cells are slightly displaced from their original location, and the sensation of metamorphopsia improves as photoreceptor cells gradually return to their original positions with time.

In contrast, contraction of the ERM causes centripetal displacement of the retinal surface, distorting the alignment of photoreceptor cells and causing metamorphopsia^20^. In both the MH and the ERM, distorted Müller cells may also contribute to the development of metamorphopsia as optic guides which transfer photons to the displaced photoreceptors as proposed in our previous publications^14,17^.

The present study had several limitations. First, it was a retrospective study with a relatively small number of cases and a short follow-up period. Second, the area of ILM removal was not measured quantitatively. Third, all the retinal thicknesses were measured manually using OCT images. Because of the retinal displacement after MH surgery, the measurement points for each retinal thickness were not identical throughout observation periods.

In conclusion, basal MH diameter is a significant predictor for metamorphopsia before and after surgery. Displaced photoreceptors appear to contribute most to generate pre- and postoperative metamorphopsia. The change of INL thickness after MH surgery also correlates with postoperative metamorphopsia, which suggests that thinning of inner retinal layer simultaneously play some role for creating a sensation of metamorphopsia.

## Data Availability

The datasets generated and analyzed during the current study are available from the corresponding author on reasonable requests.
